# Study of heterogeneity in immune responses to exposure in breast cancer: A protocol for a systematic review

**DOI:** 10.1371/journal.pone.0320498

**Published:** 2025-03-31

**Authors:** Amene Saghazadeh

**Affiliations:** Cancer Institute, Department of Pathology, Imam Khomeini Hospital Complex, Tehran University of Medical Sciences, Tehran, Iran; Xiangya Hospital Central South University, CHINA

## Abstract

**Background:**

Radiation is a treatment modality of interest in both palliative and curative settings for patients with breast cancer (BC). Despite many advances in radiation oncology, anti-radiation resistance remains a problem in mechanistic aspects. Studies show that immune cells respond to radiation in a heterogeneous manner and suggest the determinant role of these responses in the ultimate clinical classification of patients with BC as anti-radiation resistant/sensitive.

**Objective:**

A systematic review of *in vivo* and *in vitro* studies will be defined here to assess the radiation response of immune cells in BC comprehensively.

**Methods/design:**

A systematic search for *in vivo* and *in vitro* studies will collect studies that compare the radiation alone-treated and control (untreated or sham) subjects, as well as subjects treated with radiation in combination and subjects treated with radiation alone. The primary outcome of interest will be measures of the abundance of immune cell subsets. The secondary outcomes will include measures derived from co-culture assays leading to immunoregulatory roles or mechanisms proposed for the potential cross-talks between immune-BC cells that will be influenced by radiation. The review results will be primarily synthesized in a narrative and qualitative manner. Meta-analysis will be considered if there are three or more observations with data available for a specific outcome. We will use the SYRCLE “Risk of Bias” assessment tool for appraising the risk of bias in preclinical *in vivo* studies, and given the lack of standardized instruments for *in vitro* studies, we will design a checklist suitable for our specific *in vitro* research questions and publish it along with our systematic review.

## 1. Background

Breast cancer (BC) is a global health concern, with more than two million new cases reported in 2020 [1]. Projections estimate an increasing trend of burden for BC in the next two decades so that more than three million new cases will occur, and more than one million people will die from BC in 2040 [1]. Despite many advances, we witnessed in the identification of novel biomarkers for early-stage diagnosis of BC, the high rate of death from a single medical entity has been a matter of deep dispute, making us focused on the root of the problem that is resistance to cancer therapy.

Different treatment modalities for BC are available in two main modes, palliative and curative, according to whether or not distant metastasis exists [2]. Both involve multiple modalities, and radiation therapy (RT) is a shared one. RT opens radiation oncologists’ hands to consider patients’ needs carefully and prescribe different regimens to different sites at different times alone or in combination with systemic treatments accordingly. Despite that RT offers such a versatile modality with many advances in recent years, as evaluated in a review of the efficacy and safety of different radiotherapy approaches [3], patients respond to RT in very different pictures so that there is a heterogenous spectrum of radiation responses with two ends of anti-radiation sensitive to anti-radiation resistant.

Factors that contribute to the success and failure of RT include BC subtypes and RT regimens, but that is not the whole story. Studies have discovered interactions at the gene-gene [4] and cell-cell levels that mainly account for intratumor heterogeneity [5] and may, thus, be hidden drivers of the anti-radiation response of BC cells. A massive line of research is then reasonably focused on the immune cells that comprise the tumor microenvironment (TME) to trace how radiation influences non-tumor cells and how radiation-induced non-tumor cells cross-talk with tumor cells and influence their anti-radiation behavior.

Studies have identified that both innate and adaptive immune cells respond to radiation, and numerous molecular mechanisms are also postulated for their subsequent mechanism of action toward BC cells’ behavior determination. However, a significant heterogeneity across the immune cell populations of interest, pivoting around different phenotypical and functional markers used for defining the immune cells, makes it difficult to interpret the role every individual immune cell subset plays in response to radiation and then in determining BC cell behavior.

This review aims to address this problem by collecting the evidence in a comprehensive, systematic manner to answer the following questions:

A. Which markers and methods have been used for the phenotypical/functional evaluation of macrophages, monocytes, myeloid-derived suppressor cells, dendritic cells, and T cells in response to radiation for BC?
B. Is radiation able to influence different subsets of macrophages, monocytes, myeloid-derived suppressor cells, dendritic cells, and T cells in BC as defined based on answering the previous question?
C. What are the immunoregulatory roles of different subsets of macrophages, monocytes, myeloid-derived suppressor cells, dendritic cells, and T cells in response to radiation alone for BC?
D. Which treatments can amplify the favorable radiation responses of different subsets of macrophages, monocytes, myeloid-derived suppressor cells, dendritic cells, and T cells in BC?
E. Which treatments can mollify the unfavorable radiation responses of different subsets of macrophages, monocytes, myeloid-derived suppressor cells, dendritic cells, and T cells in BC?
F. How do the answers to all previous questions differ across the phenotypical/functional subsets of immune cells?

Understanding the immunology of radiation for BC in the milieu of the above-depicted systematic review of the literature is mandatory for us to see where we are and how we continue to improve.

## 2. Methods/design

We will prepare this systematic review and related protocol based on the Preferred Reporting Items for Systematic Reviews and Meta-Analyses (PRISMA) statements ([6–8]; S1 File).

### 2.1. Eligibility criteria

#### 2.1.1. Population.

This review will consider both clinical and preclinical studies for eligibility. In clinical studies, subjects with primary or secondary BC with or without metastasis will be eligible. In preclinical studies, both *in vivo* and *in vitro* experimental studies are of interest. *In vivo* studies of human and animal subjects with the same criteria as clinical studies, as well as *in vitro* studies of cell lines, models, and organoids of BC, will be included.

Animal models of BC are of four main types: induced, transplantation, genetic engineering, and spontaneous, and apply for different research purposes and answer different research questions [9]. All these models have been utilized in the design of original research in the context of our interest, i.e., radiation responses of immune cells. Regardless of model type, animal studies will be included in the systematic review so long as they meet the other eligibility criteria.

Also, both immunocompetent and immunodeficient animals are used in BC research. For this systematic review, animals of both immune states are eligible, which will enable us to compare the radiation responses of immune cells in the presence or absence of an immunosuppressive background.

In addition to high rates of metastases, contralateral BC and ipsilateral BC tumor recurrence occur in patients with median annual incidence rates of 0.6% and 0.5%, according to a systematic review [10]. In animal studies, researchers re-challenge animals or induce contralateral tumors to address whether or not immune-mediated responses to local radiation are durable and systemic. The latter, i.e., systemic effects of local radiation, is referred to as the abscopal effect [11] with both advantages and disadvantages; for their beneficial balance, the immune responses to radiation need to be finely tuned [12]. Human and animal studies investigating the radiation-induced immune cell responses in re-challenges or contralateral inductions will be included in the systematic review.

Different cell lines have been developed to approach different research questions in BC. According to a 2017 review, more than 80 cell lines are available, and these vary by hormone status, mutation status, and basic medium [13]. These cell lines derive pictures that are radiosensitive and radioresistant, resembling different clinical BC subtypes. All of them are of interest to our systematic review.

#### 2.1.2. Intervention and comparators.

**2.1.2.1. *Ionizing radiation alone:*** The intervention group will undergo RT alone. Radiation must be of ionizing type; non-ionizing radiation will be excluded. Radiation dose in the gray unit (Gy) and radiation regimens (ablative vs. hypofractionated) must be expressed.

Human studies often do not report details about radiation doses and regimens. We, therefore, include all of them in the systematic review, assuming that RT has been administered according to the available global guidelines. Both external and internal RT (intraoperative) will be included.

In multi-group studies, the comparison group will be either untreated or sham radiation control. Also, single-group studies evaluating longitudinal outcomes of interest in relation to the administration of RT will be included.

**2.1.2.2. *Ionizing radiation in combination*:** The intervention group will receive radiation in combination with at least another treatment modality. To be included in the systematic review, studies need to include at least two groups: one group undergoing RT alone and the other undergoing RT in combination. Single-group studies administering RT in combination with multi-group studies that lack the administration of RT alone will be excluded.

#### 2.1.3. Outcomes.

**2.1.3.1. *Primary*:** The primary outcomes are measures of specific immune cell subsets and defined by (1) percent, frequency, or density of cell subsets of interest and (2) concentration of markers associated with interested phenotypes or functionality.

Marker series tested for the specific cell subsets vary across studies [14], and all of them will be of interest. As well, methods of measurement are different, including immunohistochemistry, flow cytometry, enzyme-linked immunosorbent assay, polymerase chain reaction, and live imaging. All methods of measurement will be included.

**2.1.3.2. *Secondary*:** The secondary outcomes are measures of cross-talks between BC cells and immune cells elicited in response to RT. Therefore, co-culture assays, which often contain a sequence of experiments in order to conclude on a cascade of potential interactions and their effects, and related indices will be eligible for this systematic review.

### 2.2. Information sources

We will develop strategies to search for the relevant original research in the target major medical databases: Embase, PubMed, Scopus, and Web of Science. The proposed initial search strategy comprises key terms related to the population, intervention, and outcomes, which are in S2 File. The language filter is set for English (Table 1).

**Table 1:** Inclusion and exclusion criteria.

	Inclusion criteria	Exclusion criteria
**Population**	*In vivo*: human and animal subjects with primary or secondary breast cancer with or without metastasis *In vitro*: human and animal cell lines, models, and organoids of primary or secondary breast cancer with or without metastasis	
**Intervention arm**	Treated with ionizing radiation (Rad) alone or in combination with other treatment	Non-ionizing radiation
**Comparison**	In case the intervention arm is treated with Rad alone: untreated or sham control or before-after Rad treatment measurements available in single-arm studies In case the intervention arm is treated with Rad in combination, treated with Rad alone	
**Outcome**	Measurement of immune cell subsets in the peripheral blood or the tissue sections of the primary or secondary tumor, metastatic sites, lymph nodes, or spleen	
**Study design**	Case-control Cohort Clinical Preclinical Cross-sectional	Case report Case series
**Type of publication**	Original research article with full text available in English	Original research article without full text available in English Commentary Conference abstract Conference review Review Systematic review Meta-analysis Editorial Opinion Book, book chapter, or book review
**Time frame**	There is no formal period of interest for outcome measurement, and any time of measurement in relation to Rad treatment is eligible as long as it is clearly mentioned in the manuscript/declared by authors upon contact.	

No time restriction is applied, and all eligible studies from the inception to the date of the last updated search will be included in the systematic review. After retrieving all eligible studies through the database search, we will run a backward search by going through the bibliography list of included studies and checking for any further potential titles that had not been found in the database search.

The search strategy will be reviewed and updated as necessary before the final version publication to ensure inclusion of the most recent and relevant studies. If significant delays occur between original draft preparation and final manuscript publication (1 year or more), an updated search will be conducted to capture newly published literature.

### 2.3. Study selection

**Fig 1:**
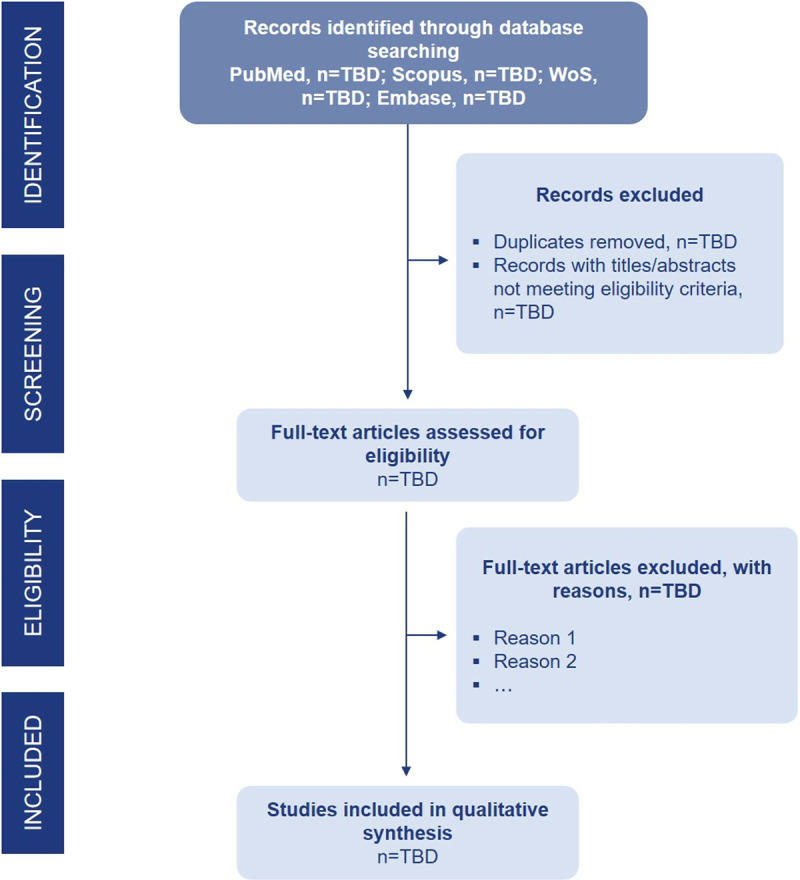
PRISMA-based study selection process. The figure presents the planned study selection process following the PRISMA (Preferred Reporting Items for Systematic Reviews and Meta-Analyses) guidelines. Since this is a protocol and the screening process has not yet been conducted, numerical values at each stage are marked as TBD (To be determined after search and screening execution). This flowchart outlines the expected steps for study identification, screening, eligibility assessment, and inclusion in the final systematic review.

Search results from the database search will be inserted into EndNote for software assistance and then handy removal of duplicates (Fig 1). Then, the title and abstract of the discrete records will be screened, and the full texts of potentially eligible records will be found for a detailed review. The full texts will be reviewed against all pre-defined inclusion/exclusion criteria. Any disagreements between reviews will be discussed to reach a consensus.

### 2.4. Data collection

We will extract data in the form of Excel worksheets. Data items will be determined and standardized after a pilot round of data extraction from eligible articles. Table 2 lists preliminary items by category. In case necessary data items for a study are missing or inconsistent in the systematic review, the corresponding author will be contacted and requested to provide additional data. A reminder with a one-week interval between the emails will follow the first request.

**Table 2:** Data items for extraction.

Category	Relevant items
**Study characteristics**	Study title, first author, year of publication, corresponding author, email address for correspondence
**Study population**	Human studies: Age, presence of comorbid conditions, BC type, BC stage, primary/secondary BC, presence of metastasis
	Animal studies: Animal type, age, gender, strain, immune status
**Breast cancer model and method of establishment**	Animal studies: Induced, transplantation, genetic engineering, spontaneous
	Cell line: Radiosensitivity status, hormone status, mutation status
**Intervention**	Radiation characteristics (source, dose, regimen) and method of administration (internal/external)
**Co-interventions**	Immunotherapy, chemotherapy, nanoplatforms, microparticles, anti-convulsants, etc.
**Immune cell measurements**	Sample (tumor tissue, peripheral blood, splenic tissues) Broad cell population of interest (monocytes, macrophages, myeloid cells, and T cells); markers tested for each specific cell subset; unit of measurement (%, IOD, mRNA, etc.); method of measurement; time post-radiation; change induced by radiation (increase, decrease, not significant); and statistical significance
**Other**	Quality assessment-related items as recommended by specific checklists for the critical appraisal of studies by type

### 2.5. Risk of bias assessment

Risk of bias will be appraised using the SYRCLE “Risk of Bias” assessment tool for preclinical *in vivo* studies [15]. In summary, this tool comprises ten domains of bias mainly related to selection bias, performance bias, detection bias, attrition bias, and reporting bias.

For *in vitro* studies, quality assessment tools are not well-defined, and scholars often develop specific ones for their own systematic review to meet the points of interest required to be critically appraised [16]. We, therefore, will design one checklist suitable for our specific research questions and publish it along with our systematic review. Key domains will include study design and experimental setup, ensuring clear hypotheses, appropriate control groups, and proper authentication of cell lines. Exposure details will be scrutinized, encompassing validation of radiation doses, characterization of chemical or biological agents, and justification regarding exposure duration and frequency. Replication details, including the number of biological and technical replicates, will be assessed alongside statistical power calculations where applicable. Outcome assessment will emphasize clearly defined primary and secondary endpoints, validation of quantification methods, and blinding in outcome evaluation to minimize bias. Data analysis and statistical reporting will be evaluated based on the appropriateness of statistical tests, transparency in data presentation, the inclusion of error bars, and clear reporting of statistical significance levels (e.g., p-values with predetermined thresholds such as p < 0.05). Additionally, reproducibility and reporting standards will be assessed by examining detailed protocol descriptions, transparency, and compliance with relevant guidelines. The finalized checklist will ensure consistency and reliability in data extraction, quality assessment, and interpretation of findings across studies.

### 2.6. Data synthesis

All the primary and secondary outcomes are of continuous type, so mean difference (MD) will be calculated with a 95% confidence interval for comparing two intervention and comparator arms or for comparing measurements at two points of time. Mean difference calculation will be done in a weighted manner or standardized based on whether or not methods/scales of measurements are the same. Effect size estimation (Cohen’s d and Hedge’s g) will be done using a random-effects model.

The systematic review will consider heterogeneity, subgroup, regression, and publication bias analyses if there are ten or more observations with data available for a specific outcome. Heterogeneity will be tested using Q and I-squared statistics. Based on data availability, subgroup, and meta-regression analyses can be done using the measurement method, BC subtype/model, radiation characteristics, etc. For publication bias, funnel plots will be visualized for any asymmetry along with statistical Egger’s and Begg’s tests. In case publication bias is present, outlier removal and trim and fill methods will be applied.

A pilot data extraction revealed significant heterogeneity across studies evaluating radiation responses of immune cells in BC due to various determinants. Main manuscripts and their related supplements often lack numerical values for the outcomes of interest. Given this, results may be synthesized narratively, and meta-analysis will be considered if there are three or more observations with available data for a specific outcome.

To address heterogeneity, qualitative evidence synthesis of eligible studies will be supplemented by grouping observations by tested markers for specific subsets, measurement methods, radiation regimens, and disease subtypes/models. This grouping allows the exploration of findings across subgroups and may identify contributing factors to heterogeneity.

This investigation will focus on the determinants explaining variations in outcomes, particularly in exploratory aspects of the review. The potential for heterogeneity to influence conclusions will be discussed in the synthesis.

We acknowledge that variability in reporting and measurement methods may limit comparability. Therefore, we will apply a flexible approach, prioritizing transparency in documenting these variations and clearly stating heterogeneity-related assumptions in the final synthesis.

### 2.7. Status and timeline of the study

This protocol describes a multi-phase project consisting of several series of studies. The first series is currently in progress. Preparatory activities, including a comprehensive literature review, are nearly complete, and subsequent stages will begin shortly. For the first series, data collection and subsequent data synthesis and result dissemination are anticipated to be completed in 2025.

This timeline reflects the project’s progressive nature and aligns with the journal’s requirements for protocols reporting on studies in progress.

### 2.8. Ethics approval and consent to participate

This study is a protocol for a systematic review and does not involve human participants or personal data collection. Therefore, ethics approval and consent to participate are not required.

## 3. Summary

To our knowledge, we will perform this comprehensive systematic review for the first time. Systematic review and meta-analysis studies have been published on the efficacy of different types of radiation therapy, e.g., partial- vs. whole-breast or hypofractionated vs. conventional fractionated, for different BC subtypes and metastases in terms of clinically relevant outcomes, e.g., survival, recurrence, and side effects [17–21]. Yet, there is a lack of any formal systematic review on the immune cells-mediated mechanism of action of radiation that is largely, if not wholly, responsible for the clinical behavior of BC patients treated with radiation. Heterogeneity in clinical responses is the mirror of heterogeneity in immune cell responses, and our systematic review will attempt to approach the problem by defining heterogeneity assemblies of immune cell responses playing a role in anti-radiation responses.

## Supporting information

S1 File PRISMA-P checklist.docx. (DOCX)

S2 File PubMed search strategy. (DOCX)
