# Supplementary material for: Study of heterogeneity in immune responses to exposure in breast cancer: A protocol for a systematic review
Source: PLoS One. 2025 Mar 31;20(3):e0320498. doi: 10.1371/journal.pone.0320498 (PMC11957354; doi:10.1371/journal.pone.0320498)
Supplement: S2 File — (DOCX) [file pone.0320498.s002.docx]

**Study of Heterogeneity in Immune Responses to Exposure in Breast Cancer:**

**A Protocol for a Systematic Review**

**Additional file 2: PubMed search strategy**

1. **Helper T cells and cytotoxic T cells**

((lymphocyte*[Title/Abstract] AND (english[Filter])) OR (t cell*[Title/Abstract] AND (english[Filter])) AND (english[Filter])) AND (((Radiation[Title/Abstract] OR Radiotherapy*[Title/Abstract] OR Irradiation[Title/Abstract] OR Radiosensit*[Title/Abstract] OR Radioresist*[Title/Abstract] AND (english[Filter])) AND (Neoplasm*[Title/Abstract] OR cancer*[Title/Abstract] OR tumor*[Title/Abstract] OR tumour*[Title/Abstract] OR carcinoma*[Title/Abstract] OR adenocarcinoma*[Title/Abstract] AND (english[Filter]))) AND (breast[Title/Abstract] OR mammary[Title/Abstract] AND (english[Filter])) AND (english[Filter]))

1. **Dendritic cells**

(("Radiation"[Title/Abstract] OR "radiotherapy*"[Title/Abstract] OR "Irradiation"[Title/Abstract] OR "radiosensit*"[Title/Abstract] OR "radioresist*"[Title/Abstract]) AND "english"[Language] AND (("neoplasm*"[Title/Abstract] OR "cancer*"[Title/Abstract] OR "tumor*"[Title/Abstract] OR "tumour*"[Title/Abstract] OR "carcinoma*"[Title/Abstract] OR "adenocarcinoma*"[Title/Abstract]) AND "english"[Language]) AND (("breast"[Title/Abstract] OR "mammary"[Title/Abstract]) AND "english"[Language]) AND "english"[Language] AND ("dendritic cell*"[Title/Abstract] AND "english"[Language])) AND (english[Filter])

1. **Macrophage and myeloid cells**

(((monocyte*[Title/Abstract] AND (english[Filter])) OR (myeloid*[Title/Abstract] AND (english[Filter]))) OR (Macrophage*[Title/Abstract] AND (english[Filter])) AND (english[Filter])) AND (((Radiation[Title/Abstract] OR Radiotherapy*[Title/Abstract] OR Irradiation[Title/Abstract] OR Radiosensit*[Title/Abstract] OR Radioresist*[Title/Abstract] AND (english[Filter])) AND (Neoplasm*[Title/Abstract] OR cancer*[Title/Abstract] OR tumor*[Title/Abstract] OR tumour*[Title/Abstract] OR carcinoma*[Title/Abstract] OR adenocarcinoma*[Title/Abstract] AND (english[Filter]))) AND (breast[Title/Abstract] OR mammary[Title/Abstract] AND (english[Filter])) AND (english[Filter]))
